# The Toluene *o*-Xylene Monooxygenase Enzymatic Activity for the Biosynthesis of Aromatic Antioxidants

**DOI:** 10.1371/journal.pone.0124427

**Published:** 2015-04-27

**Authors:** Giuliana Donadio, Carmen Sarcinelli, Elio Pizzo, Eugenio Notomista, Alessandro Pezzella, Carlo Di Cristo, Federica De Lise, Alberto Di Donato, Viviana Izzo

**Affiliations:** 1 Department of Biology, University of Naples Federico II, Via Cinthia I, 80126, Napoli, Italy; 2 Department of Chemical Sciences, University of Naples Federico II, via Cinthia I, 80126, Napoli, Italy; 3 Department of Sciences and Technologies, University of Sannio, Via Port'Arsa, 11, Benevento, Italy; 4 Department of Medicine and Surgery, University of Salerno, via S. Allende, 84081, Baronissi (SA), Italy; Islamic Azad University-Mashhad Branch, Mashhad, Iran, IRAN, ISLAMIC REPUBLIC OF

## Abstract

Monocyclic phenols and catechols are important antioxidant compounds for the food and pharmaceutic industries; their production through biotransformation of low-added value starting compounds is of major biotechnological interest. The toluene *o*-xylene monooxygenase (ToMO) from *Pseudomonas* sp. OX1 is a bacterial multicomponent monooxygenase (BMM) that is able to hydroxylate a wide array of aromatic compounds and has already proven to be a versatile biochemical tool to produce mono- and dihydroxylated derivatives of aromatic compounds. The molecular determinants of its regioselectivity and substrate specificity have been thoroughly investigated, and a computational strategy has been developed which allows designing mutants able to hydroxylate non-natural substrates of this enzyme to obtain high-added value compounds of commercial interest. In this work, we have investigated the use of recombinant ToMO, expressed in cells of *Escherichia coli* strain JM109, for the biotransformation of non-natural substrates of this enzyme such as 2-phenoxyethanol, phthalan and 2-indanol to produce six hydroxylated derivatives. The hydroxylated products obtained were identified, isolated and their antioxidant potential was assessed both *in vitro*, using the DPPH assay, and on the rat cardiomyoblast cell line H9c2. Incubation of H9c2 cells with the hydroxylated compounds obtained from ToMO-catalyzed biotransformation induced a differential protective effect towards a mild oxidative stress induced by the presence of sodium arsenite. The results obtained confirm once again the versatility of the ToMO system for oxyfunctionalization reactions of biotechnological importance. Moreover, the hydroxylated derivatives obtained possess an interesting antioxidant potential that encourages the use of the enzyme for further functionalization reactions and their possible use as scaffolds to design novel bioactive molecules.

## Introduction

In living organisms, aerobic metabolic processes such as respiration and photosynthesis cause the generation of reactive species of either oxygen (ROS) or nitrogen (RNS) in specific organelles such as mitochondria, chloroplasts, and peroxisomes. Both ROS and RNS share a well-documented role in stimulating physiological events such as signaling and cell differentiation. However, these molecules are also highly unstable and tend to acquire electrons from nucleic acids, lipids, proteins, carbohydrates causing a sequence of chain reactions that are responsible for cellular damage [1,2]. The correct balance inside the cell between the prooxidant action of both ROS and RNS and the antioxidant activity of either enzymatic (such as superoxide dismutase, catalase, glutathione peroxidase and glutathione reductase) or non-enzymatic (such as vitamin C, vitamin E, selenium, zinc, taurine, hypotaurine, glutathione, β-carotene) systems is indeed a very delicate equilibrium which is maintained through the activity of a complex array of molecular mechanisms [1,3].

Evidence has been accumulating which shows that oxidative stress, the condition deriving from an imbalance between prooxidants and antioxidants in the cell, might represent a common element for the onset of several pathologies such as cancer, neurodegenerative and cardiovascular diseases, and the occurrence of complications in diabetes [4,5].

The quest for novel natural and synthetic antioxidants has undoubtedly drawn the attention of the scientific community in recent years. In this context, several antioxidant pharmacophores have been identified, among which phenols and catechols have been given much attention as they are very susceptible to oxidation by acting as electron donors [6, 7].

Phenols are endowed with the property of suppressing or delaying spontaneous autoxidation of organic molecules and might be useful to prevent *in vivo* the consequences of the autoxidation of biomolecules [6,8]. Thus, it is not surprising that fruits and vegetables, which are characterized by a high content of phenolic compounds such as phenolic acids and flavonoids, have been so far the main suppliers of molecules analyzed as potential antioxidant drugs, and their consumption in a healthy and varied diet has been demonstrated to significantly reduce the risk of cancer [9,10–13]. In addition, synthetic phenolic antioxidants such as butylated hydroxyanisole (BHA), butylated hydroxytoluene (BHT), tert-butylhydroquinone (TBHQ) and propyl gallate (PG) have been also studied and used, although the use of BHA and BHT has been restricted by legislative rules due to uncertainties concerning their toxic and carcinogenic effects [6].

Despite all the interest towards phenolic antioxidants, there are still many issues to unravel concerning their biological activity. The analysis of kinetic and thermodynamic data available so far in the literature indicates that the efficiency of phenols as inhibitors of the autoxidation of organic matter depends on a variety of factors, and this becomes undoubtedly challenging in complex systems such as living organisms [6].

Other antioxidant pharmacophores have been identified, among which catechols, 1,2-dihydroxybenzenes, have been given much attention [7]. It should be noted that the Comprehensive Medicinal Chemistry (CMC) database shows the presence of many drugs containing the catechol moiety for which several pharmacological effects have been described [14,7]. For these molecules, it is commonly accepted that their antioxidant effect depends on the fact that the semiquinone radical derived from H-atom donation of catechol can be stabilized by both an intramolecular hydrogen bond and by the electron-donating properties of the *ortho*-OH [15–16]. Thus, the chemical versatility of both phenols and catechols and the numerous pharmacological effects described for some of their derivatives, encourage their use as scaffolds to design novel bioactive molecules where the different nature of the substituents bound to the aromatic ring may influence the reactivity of these molecules towards oxidizing agents, their pharmacokinetics, and their tissue and cellular distribution [14,7].

However, the synthesis of substituted phenols and catechols by chemical methods is often complex and may involve severe reaction conditions, resulting in low yields and the formation of racemic mixtures [17,18]. These complications have boosted the research for the development of biotransformations, also known as bioconversions, which make use of the metabolic versatility of either purified enzymes or whole microorganisms, for the selective hydroxylation of commercially available, low-added-value, aromatic precursors [19].

The toluene *o*-xylene monooxygenase (ToMO) from *Pseudomonas* sp. OX1 is a bacterial multicomponent monooxygenase (BMM) [19–23] that has been extensively characterized in the last decade from both a biochemical and a biotechnological point of view [24–26]. The molecular determinants of its regioselectivity and substrate specificity have been thoroughly investigated, and a computational strategy has been developed which analyzes the interactions between the active-site residues of its hydroxylase component ToMOA and the substrates [26–31]. Recently, mutants of the ToMO multicomponent system were used to produce tyrosol (4-hydroxyphenylethanol, bearing a phenol moiety) and hydroxytyrosol (3,4-dihydroxyphenylethanol, bearing a catechol moiety), strong antioxidants commonly found in extra virgin olive oil [32–36,13], using a cheap and commercially available aromatic precursor such as 2-phenylethanol (S1 Fig) [18]. The results obtained showed the great versatility of ToMO and suggested the potential use of this enzyme for the hydroxylation of other non-natural aromatic substrates to produce antioxidant phenols and catechols of interest for both the food and pharmaceutical industries.

In the present study we have used the recombinant ToMO system, expressed in strain JM109 of *E*.*coli*, to hydroxylate three commercially available aromatic monocyclic molecules that are non-natural substrates of the enzyme, to obtain mono- and dihydroxylated derivatives analogous to natural antioxidants tyrosol and hydroxytyrosol, but with some structural differences that might influence their antioxidant potential.

**Fig 1:**
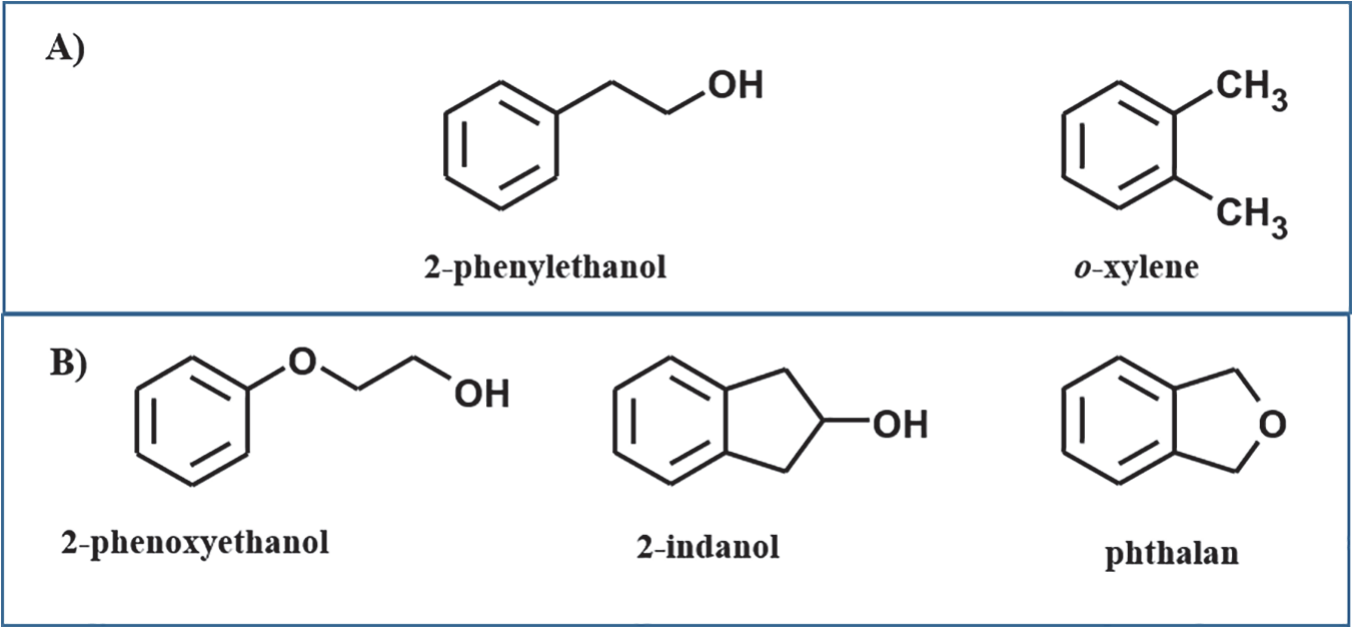
Starting substrates. **(A)** Structures of 2-phenylethanol and *o*-xylene. **(B)** Substrates used for the ToMO-catalyzed biotransformations presented in this study.

The three aromatic molecules used in this study as starting compounds are benzene derivatives bearing one or two substituents in *ortho* (Fig 1B):

- 2-phenoxyethanol, is a benzene derivative with a-O-(CH_2_)_2_-OH substituent bound to the aromatic ring. This compound differs from 2-phenylethanol (Fig 1A), the starting substrate used for the biosynthesis of tyrosol and hydroxytyrosol [18] (S1 Fig), for the presence of an additional oxygen atom directly linked to the aromatic ring;
- 2-indanol, is similar to 2-phenylethanol but with an additional methylene group, linking the ethanol moiety to the ring, which makes the structure more rigid;
- phthalan, which differs from *o*-xylene, a natural substrate of ToMO (Fig 1A), for the presence of an oxygen atom bridging the two methyl substituents.

Phenols and catechols obtained from the hydroxylation of these compounds were isolated, purified and identified by NMR and mass spectrometry analysis. Their antioxidant potential was first assessed *in vitro* using the DPPH chemical assay [37–38], and then on the rat cardiomyoblast cell line H9c2 [39–40] to evaluate their effect during a mild oxidative stress induced by sodium arsenite (SA). Exposure to SA inhibits protein synthesis in eukaryotic cells and activates multiple stress signaling pathways such as the generation of what is usually referred to as “stress granules” [4,41–43]. Confocal microscopy analysis suggested that in stressed H9c2 cells, pretreatment with optimal concentrations of the hydroxylated compounds resulted in a lower percentage of cells containing stress granules, thus highlighting a potential antioxidant activity of these molecules in the cellular *milieu*, and paving the way to their future functionalization to increase and diversify their biological activities.

## Materials and Methods

### Generals

Bacterial cultures, plasmid purifications, and transformations were performed according to Sambrook et al [44]. *E*. *coli* strain JM109 was from Novagen. Plasmid pGEM3Z/Tou *wt* and mutant E103G/F176A were prepared as already described [18]. 2-phenoxyethanol, phthalan, and 2-indanol were purchased from Sigma-Aldrich. Water and methanol used for HPLC experiments were from Romil. Formic acid was from Carlo Erba.

### Docking of substrates in ToMOA active site

Reaction intermediates were docked into the active site of ToMO by using the Monte Carlo energy minimization strategy as previously described [18], except that the 2010 version of the ZMM software was used, and the dielectric constant was calculated using the default option of this version. The PDB files for the initial manually generated complexes and the ZMM instruction files containing the lists of mobile residues, constraints, and parameters used during calculations are available upon request.

### ToMO-catalyzed bioconversion of 2-phenoxyethanol, phthalan and 2-indanol: isolation and purification of the reaction products

Cells of *E*. *coli* strain JM109 transformed with either plasmid pBZ1260 or variant E103G/F176A, were routinely grown in LB to which ampicillin was added to a final concentration of 100 μg/mL (LB/amp). Several well-isolated colonies were picked from LB/amp agar plates, inoculated into 12.5 mL of LB/amp in a sterile 50 mL Falcon Tube and incubated in constant shaking at 37°C up to 0.6 OD_600_. The preinoculum was diluted 50-fold in two 500 mL Erlenmeyer flasks containing each 250 mL of LB/amp, and incubated under constant shaking at 37°C up to 0.7–0.8 OD_600._ Expression of the recombinant ToMO proteins was induced with 0.2 mM IPTG at 37°C and in the presence of 0.2 mM Fe(NH_4_)_2_(SO_4_)_2_; this latter was necessary for the assembly of the iron-containing catalytic center of the recombinant ToMO. After 1 hour of induction, cells were collected by centrifugation (1,880 x g for 15’ at 4°C), resuspended in M9 medium containing 0.4% glucose (M9-G) at a final concentration of 6 OD_600_ and incubated with 2 mM of either 2-phenoxyethanol, phthalan, or 2-indanol dissolved in methanol (final methanol concentration was 2%). After a 16h incubation at 28°C under constant shaking at 230 rpm, cells were collected by centrifugation at 1,880 x g for 15’ at 4°C; an aliquot of the cell-free supernatants was loaded on HPLC to check for the presence of hydroxylated products before their purification (S2 Fig). To this purpose, an HPLC system equipped with a Waters 1525 binary pump coupled to a Waters photodiode array detector was used. Substrates and hydroxylated derivatives were separated using a Ultrasphere C_18_ reverse-phase column (4.6 x 250 mm; particle size 10 μm, pore size 100 Å). Separation was carried out at a flow rate of 1 mL/min by using a two-solvent system consisting of a 0.1% formic acid solution in water (solvent A) and a 0.1% formic acid solution in methanol (solvent B). Compounds were separated using a 3-min isocratic elution with 10% solvent B, followed by a 20-min linear gradient from 10 to 75% solvent B and an isocratic 5-min step at 98% solvent B.

For the purification of the hydroxylated products identified by analytical HPLC, cell-free supernatants, prepared as described above, were extracted twice with ethyl acetate added in a 1:2 ratio with respect to the aqueous phase. The organic phase was recovered, dried with a Rotavapor apparatus, and the pellet was dissolved in 100% methanol and stored at -20°C. The sample was then diluted to a final 10% concentration of methanol and loaded on HPLC using an Econosil C18 reverse-phase semi-preparative column (10 x 250 mm, particle size 10 μm, pore size 100 Å). Separation was carried out at a flow rate of 2 mL/min by using a two-solvent system consisting of a 0.1% formic acid solution in water (solvent A) and a 0.1% formic acid solution in methanol (solvent B). Compounds were separated using a 10-min isocratic elution with 10% solvent B, followed by a first 10-min linear gradient from 10 to 40%, a second 10-min linear gradient from 40% to 75%, a third 5-min gradient from 75% to 90%, an isocratic 10-min step at 90% of solvent B and a final 15-min step at 10% of solvent B. Each peak of interest was individually collected, concentrated, and stored at -20°C.

### Identification by NMR and mass spectrometry analysis

For NMR analysis, aliquots of the purified cell-free supernatants, prepared as described in the previous paragraph and fractionated by HPLC, were lyophilized, and directly used for NMR analysis in deuterated methanol. ^1^H NMR spectra were recorded at 600, 400, or 300 MHz, and ^13^C NMR spectra were recorded at 75 MHz. 1H and 13C distortionless enhancement by polarization transfer heteronuclear single-quantum correlation and 1H and 13C heteronuclear multiple-quantum correlation (HMBC) experiments were run at 600 and 400 MHz, respectively, on instruments equipped with a 5 mm 1H/broadband gradient probe with inverse geometry using standard pulse programs. The HMBC experiments used a 100-ms long-range coupling delay. Chemical shifts are reported in δ values (ppm) downfield from tetramethylsilane. Regioisomers were distinguished according to the spin-coupling pattern in the aromatic region of the ^1^H NMR spectra (S3 Fig). Compound eluting in peak **1** (S2 Fig), indicated from now on as compound **1**, showed only two signals featuring a doublet shape as a consequence of the ortho-type coupling. On this basis, the direct attribution of the symmetric para-substituted structure to compound **4HEP**, recognized as the 4-(2-hydroxyethoxy)phenol, was allowed. In the case of compounds eluting in peaks **2** and **3** (compounds **2** and **3**) a more complex pattern in the aromatic region of the ^1^H NMR spectra was present, suggesting, respectively, a meta and an ortho substitution of the aromatic rings (S3 Fig). The characterization of compounds **2** and **3** as 3-(2-hydroxyethoxy)phenol (**3HEP**) and 2-(2-hydroxyethoxy)phenol (**2HEP**), respectively, resulted from the ratios of shielded and unshielded signals. Indeed, the ^1^H integration for compound **3HEP** is accounted with the two H atoms in ortho (H_o_) and the one H atom in para (H_p_) to the OH group. For compound **2HEP**, the ratio H_o_/H_p_ is 1 which is coherent with the presence of only two shielded H atoms, the one in ortho and one in para to the OH group.

HR ESI+/MS spectra (S4 Fig) were obtained in 0.5% Formic Acid/methanol 1:1 v/v and were recorded on a Agilent 1100 LC/MSD/ESI apparatus.

### Evaluation of the concentration of the hydroxylated derivatives of 2-phenoxyethanol, phthalan and 2-indanol

To evaluate the concentration of the compounds obtained from the ToMO-catalyzed hydroxylation reaction we selected, for each of them, a similar compound whose UV-vis spectrum and corresponding extinction coefficient was available in the literature (S5 Fig). All the spectroscopic data used for calculations were obtained from the “Organic Electronic Spectral Data” book series (*Analitical chemistry*. *58*. *1886*. *G*. *Norwitz et al Wiley eds*).

### DPPH assay

2,2-diphenyl-1-picrylhydrazyl (DPPH) is a stable free radical with λ_max_ at 515 nm (purple, ε_515_ in methanol = 12 mM^-1^cm^-1^). The neutralization of the DPPH radical is responsible for the disappearance of the absorbance at 515 nm and is commonly used as a readout of radical scavenging of an antioxidant compound [37–38]. The reaction mixture contained, in a final volume of 1 mL of methanol, different concentrations of our hydroxylated compounds and 0.1 mM of a freshly prepared DPPH solution in methanol. It is important to note that, as recommended by Kedare and coauthors [38] the initial DPPH concentration should give an absorbance ≤ 1.0. The reaction was allowed to proceed at RT until completion, and was followed at λ = 515nm.

### Cell cultures and treatments

H9c2 cells (Rat Embryonic Myocardium Cells, CRL-1446) were purchased from American Type Culture Collection (ATCC) and grown in Dulbecco’s modified Eagle medium (DMEM) supplemented with 10% fetal bovine serum (FBS), 4 mM L-glutamine, 1% v/v penicillin/streptomycin solution (100 U/mL) and 1mM sodium pyruvate (Euroclone, Milano, Italy) at 37°C in 5% CO_2_ incubator. Oxidative stress was induced by adding sodium arsenite (Sigma Aldrich), freshly prepared in stock aqueous solutions and added to the conditioned medium at a final concentration of 0.25 mM.

### MTT assay

H9c2 cells were seeded on 96-well plates at a density of 2.5×10^3^ cells/well in 0.1 mL of DMEM 24 h prior to the treatment with the different compounds. As control, cells were incubated with buffer diluted in medium.

After 72 h of incubation, cell viability was evaluated by MTT assay, adding tetrazolium MTT (3-(4,5-dimethylthiazol-2-yl)-2,5-diphenyltetrazolium bromide) diluted at 0.5 mg/mL in DMEM without red phenol (0.1 mL/well). After 4 h of incubation at 37°C, the resulting insoluble formazan salts were solubilized in 0.04 M HCl in anhydrous isopropanol, and quantified by measuring the absorbance at λ = 570nm, using an automatic plate reader spectrophotometer (VICTOR^3^ Multilabel Counter; Perkin Elmer, Shelton, CA, USA). Cell survival was expressed as means of the percentage values compared to control. Standard deviations were always < 5% for each repeat (values in quadruplicate of at least three experiments).

The hydroxylated derivatives of 2-phenoxyethanol, phthalan and 2-indanol were used at a concentration close to their EC_50_ value (5 to 10 μM higher) obtained from the DPPH assay. When the EC_50_ was not available, as in the case of tyrosol, **3HEP**, **DHI**, and **DHiBF** (S4 Fig), two arbitrary concentrations were initially tested, 40 and 80 μM. As results obtained with these two concentrations were comparable, the lower concentration (40 μM) was used to test the potential antioxidant activity of these compounds in the following experiments such as the apoptosis assay and the analysis of stress granules.

The non-hydroxylated precursors, when assayed, were used at a concentration equal to the higher concentration used for any of their hydroxylated derivatives. In conclusion, the concentrations used in the experiments with H9c2 cells presented in the “Results and Discussion” section of this manuscript were: 25 μM for hydroxytyrosol, 40 μM for phenylethanol, tyrosol, **4HEP**, **3HEP**, phthalan, **DHiBF**, 2-indanol, **DHI**, **THI**, and 90 μM for 2-phenoxyethanol and **2HEP** (S4 Fig).

### EB and AO staining assay

Apoptotis was evaluated by ethidium bromide (EB) and acridine orange (AO) staining of nuclei [45]. AO is a fluorescent DNA binding dye that permeates all the cells staining the nuclei green, whereas EB is only taken up by the cells when cytoplasmic membrane integrity is compromised, staining apoptotic nuclei red. H9c2 cells were seeded in 6-well plates at a density of 1.5 ×10^5^ cells/well in 2 mL of DMEM and grown up to nearly 60% confluence. The cells were then incubated at 37°C for 3 h in the presence of the tested compounds. Afterwards, the SA-induced oxidative stress (0.25 mM for 90 min) was performed. Cells were trypsinized, collected by centrifugation, and washed with ice-cold PBS. Cells were re-suspended in 50 μL of PBS containing the EB/AO dye mixture (5 μg/mL) and incubated at 37°C for 20 min. Stained cells were placed on a microscope slide and covered with coverslips. Images were taken at a 100 X magnification. Both apoptotic (red) and live (green) cells were counted in five microscopic fields for each sample, to estimate the percentage of apoptotic cells.

### Immunofluorescence analysis and confocal microscopy

H9c2 cells were seeded onto coverslips in 24-well plates at a density of 2.5×10^4^ cells/well in 0.5 mL of DMEM and grown for 48 h, up to nearly 60% confluence. Cells were incubated at 37°C for 3 h with either the starting aromatic compounds or their hydroxylated derivatives. Afterwards, an incubation with 0.25 mM SA for 90 min was performed. As negative and positive controls of the stress effect, cells were treated with either buffer or 0.25 mM SA, respectively, in the absence of any exogenous compound. Cells were then fixed in 4% paraformaldehyde in PBS 1X at room temperature for 15 min and rinsed three times with PBS 1X. Quenching of free aldehydic sites of the fixative was performed by adding 50 mM NH_4_Cl in PBS 1X, at room temperature for 15 min. After washing with PBS 1X, cells were permeabilized using 0.1% Triton X-100 in PBS 1X at room temperature for 30 min, then rinsed in PBS 1X for three times. After incubating the cells in a blocking solution consisting of 5% bovine serum albumin (BSA) in PBS 1X at room temperature for 1 hour under constant shaking, PABP monoclonal antibody (Sigma Aldrich), a specific marker used to evidence the presence of stress granules in the cell, was used at 2 μg/mL in blocking buffer at 4°C overnight. After washing in PBS 1X, cells were incubated with secondary antibody Cy3 conjugated goat anti-mouse F(ab’)2 (1:1,000 dilution in blocking buffer) (Jackson Immuno Research Laboratories, UK), at room temperature for 1 hour. For nuclear counterstaining, cells were incubated with DAPI, diluted at 0.1μg/mL in PBS 1X, at room temperature for 10 min. After washing, coverslips were mounted in 50% glycerol in PBS 1X. Images were captured using a Zeiss confocal laser-scanning microscope LSM 510 using suitable lasers. Image analyses on stress granules was performed using “*Stress granule counter*” and “*Analyze particles*” ImageJ (http://rsbweb.nih.gov/ij/index.html) plugins. Nuclear counterstaining was used to count the total number of cells. Statistical analyses were performed using GraphPad (Prism) software ver. 5.0.

## Results and Discussion

### ToMO-catalyzed hydroxylation of 2-phenoxyethanol, phthalan and 2-indanol

The aromatic substrates used in this study (Fig 1B) were used without any further purification. For each compound the corresponding UV-vis spectrum was recorded after dilution in methanol; λ_max_ of 2-phenoxyethanol, phthalan and 2-indanol were respectively 269.7, 270.9 and 272.1 nm. ToMO-catalyzed biotransformation of these aromatic substrates, used at a final concentration of 2 mM in the incubation medium M9-G, was performed by using recombinant cells of *E*.*coli*, strain JM109, transformed with the plasmid coding for the complete ToMO *wt* enzymatic system, as described in detail in Materials and Methods.

After incubation for different time intervals (0, 30 min, 1 h, and 16 h) at 30°C under constant shaking (220 rpm), samples were withdrawn, cells were collected by centrifugation, and an aliquot of the exhausted medium was loaded on HPLC to check for the presence of hydroxylated products (Materials and Methods, and S2 Fig). As evident from the chromatographic profiles obtained, ToMO *wt* was able to hydroxylate 2-phenoxyethanol and phthalan. Three different peaks eluting respectively at 10.4 min (compound **1**, λ_max_ = 287.5 nm), at 12.6 min (compound **2**, λ_max_ = 273.5nm) and 15.2 min (compound **3**, λ_max_ = 275.3nm) were evident when using 2-phenoxyethanol as the substrate (Panel A in S2 Fig). Formation rates of compounds **1**, **2** and **3** were of 0.89(±0.04), 1.15(±0.20) and 0.510(±0.001) μM min^-1^, respectively. Compounds **1**, **2** and **3** accounted respectively for the 37.8(± 3.1), the 43.8(±1.3) and the 18.3(±1.8)% of the total yield of product which was of 15.3(±1.3)% (total μM products/μM substrate) and was calculated after 2h of incubation, the maximum time at which the products yield was still proportional to the time of incubation (data not shown). When using phthalan as substrate a single peak of product was eluted at 14.3 min (compound **4**, λ_max_ = 279.2 nm) (Panel B in S2 Fig) with a formation rate of 1.79(±0.03) μM min^-1^ and a yield of 10.8(±0.2)% after 2h of incubation. No evident hydroxylation product was instead observed when the substrate used for the ToMO-catalyzed bioconversion was 2-indanol (data not shown).

**Fig 2:**
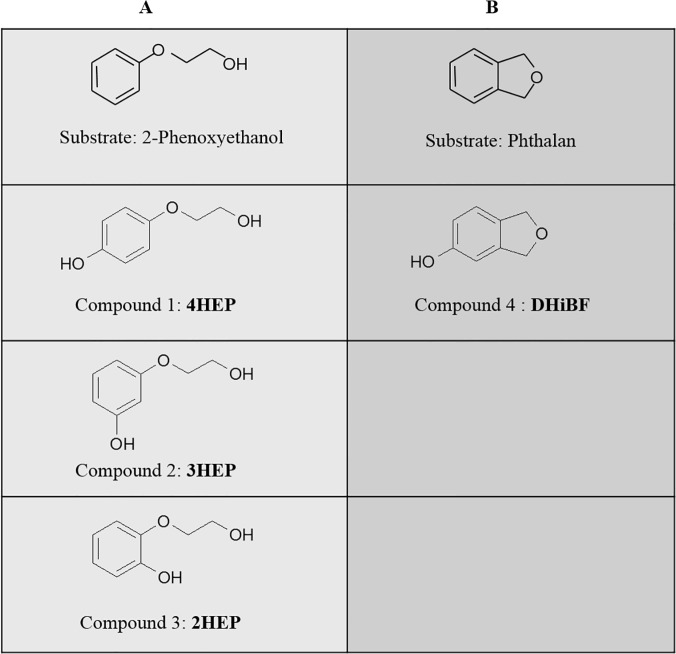
Hydroxylated products. Starting substrates and corresponding hydroxylated products obtained through ToMO-catalyzed biotransformation of (**A**) 2-phenoxyethanol and (**B**) phthalan.

The exhausted media obtained from the ToMO *wt*-catalyzed bioconversion of 2-phenoxyethanol and phthalan were further purified through organic extraction, as described in detail in Materials and Methods. Samples were then loaded on a semi-preparative HPLC C-18 column. Each peak was manually collected, lyophilized and analyzed by NMR and mass spectrometry analysis (Materials and Methods, and S3 and S4 Figs). Fig 2A and 2B show the starting compounds 2-phenoxyethanol and phthalan and the corresponding products identified through these analyses.

### Selection of an efficient catalyst for the hydroxylation of 2-indanol

The conversion of 2-phenoxyethanol to a mixture of the three mono-hydroxylated products is not surprising as this result is very similar to what previously observed in the case of 2-phenylethanol [18]. As for phthalan, the most similar substrate we have previously tested, *o*-xylene is converted to a mixture of 3,4-dimethylphenol (80%) and 2,3-dimethylphenol (20%) [27]. We have previously suggested that this result is due to the fact that the site for the ortho substituents is more hindered than the site for the para substituents [26]. Likely, in the case of phthalan the steric hindrance caused by the additional oxygen atom (Fig 1B) further disfavors the proximal hydroxylation of this compound, thus providing exclusively the hydroxylation product corresponding to 3,4-dimethylphenol *i*.*e*. 1,3-dihydro-5-hydroxyisobenzofuran (**DHiBF),** (Fig 2B and S4 Fig).

**Fig 3:**
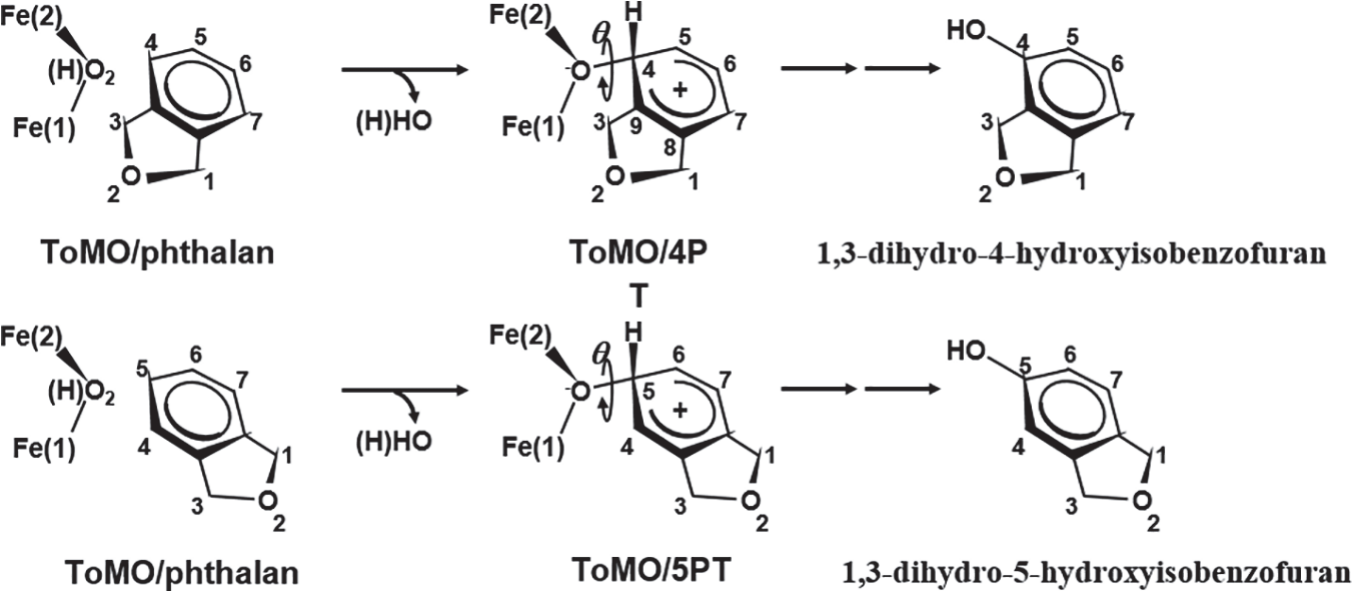
Docking of intermediates in ToMO active site. Docking of the two possible arenium intermediates (4PT and 5PT) deriving from phthalan in the active site of ToMOA. In the case of 4PT, *θ* is the torsion angle Fe(2)-O-C4-C9, whereas, in the case of 5PT, *θ* is the torsion angle Fe(2)-O-C4-C5. Similar arenium intermediates can be hypothesized for the hydroxylation of 2-indanol.

In order to identify a ToMO variant able to hydroxylate 2-indanol, we have analyzed the interaction of 2-indanol and phthalan (this latter considered as our positive control) with the active site of ToMO using a Monte Carlo modelling strategy previously applied to ToMO natural substrates and to 2-phenylethanol [26, 18]. Briefly, this strategy is based on the docking of a crucial intermediate of the hydroxylation reaction *i*.*e*. the “arenium intermediate”. By comparing the geometrical parameters for any desired complex ToMO (ToMO-variant)/intermediate to those found for the complex ToMO *wt*/arenium intermediate for para-hydroxylation of the optimal substrate toluene (the “reference complex”), it is possible to predict if an aromatic compound will be a good substrate and which product(s) will be produced. As described previously [26,18], two geometrical parameters defining the orientation of the arenium ring with respect to the diiron cluster were used for the comparison: (i) the distance between the carbon atom of the substrate that accepts the oxygen atom from the diiron cluster during the hydroxylation reaction (herein called C_n_) and the C4 atom of the reference complex, and (ii) Δ*θ*, a parameter that measures the angle deviation of the plane of the arenium ring in a ToMO mutant/intermediate complex with respect to the plane of the arenium ring in the reference structure. *θ* is the torsion angle Fe2-O-C_n_-C_m_ where C_m_ is the carbon atom of the substrate adjacent to C_n_ closer to the substituent bound to the benzene ring (Fig 3). In the reference structure, θ angle (Fe2-O-C4-C3) is 103.6°. Previously [26, 18] we have demonstrated that C_n_-C4 distances lower than about 0.2 Å, and Δ*θ* values in the range -10°/+5° are predictive of high *k*_cat_ values (similar to or even higher than those measured for ToMO *wt* on its natural substrate toluene).

Data in Table 1 show that in the case of phthalan only the arenium intermediate leading to 1,3-dihydro-5-hydroxyisobenzofuran (**DHiBF**) docks in an orientation very similar to that of the reference structure suggesting that this isomer can be efficiently produced by ToMO. On the contrary, the arenium intermediate leading to 1,3-dihydro-4-hydroxyisobenzofuran adopts an orientation very different from that of the reference intermediate described above. According to our model this would indicate poor or none production of the 4-hydroxy isomer in spite of a non-covalent binding energy value (ncBE in Table 1) more negative than that of the 5-hydroxy isomer. Overall, data in Table 1 allow to predict that ToMO can convert phthalan only to 1,3-dihydro-5-hydroxyisobenzofuran in agreement with the experimental finding. In the case of 2-indanol both arenium intermediates 24I and 25I leading to the two possible products, 2,4-dihydroxyindan and 2,5-dihydroxyindan respectively, dock into the active site with orientations very different from that adopted by the natural substrate toluene. An accurate examination of the complex ToMO/25I showed that the steric hindrance of phenylalanine 176 prevents the correct accommodation of the intermediate in a manner similar to that previously described for the *meta*- and *para*-hydroxylation of phenylethanol [18]. However, in the case of 2-indanol the presence of the additional methylene group and the rigidity caused by the ring structure markedly increase the deviation from the ideal geometry (Table 1) in agreement with the fact that ToMO slowly converts phenylethanol to tyrosol isomers but seems to be almost completely inactive on 2-indanol.

**Table 1:** Geometrical parameters and non covalent binding energies (ncBE) of the complexes between ToMO or E103G/F176A-ToMO and the arenium intermediates for the phthalan/1,3-dihydro-4-hydroxyisobenzofuran (4PT), the phthalan/1,3-dihydro-5-hydroxyisobenzofuran (5PT), the 2-indanol/2,4-dihydroxyindan (24I) and the 2-indanol/2,5-dihydroxyindan (25I) reactions.

Arenium intermediate	Geometrical parameters	ToMO	
		wild type	E103G/F176A
	**C4-C4 (Å)**	0.42	- ^*a*^
**4PT**	**Δ*θ* (°)**	+36.7	-
	**ncBE (kcal mol** ^**-1**^ **)**	-42.4	-
	**C4-C5 (Å)**	0.25	-
**5PT**	**Δ*θ* (°)**	-6.7	-
	**ncBE (kcal mol** ^**-1**^ **)**	-33.5	-
	**C4-C4 (Å)**	0.49	0.45
**24I**	**Δ*θ* (°)**	+28.8	+35.6
	**ncBE (kcal mol** ^**-1**^ **)**	-40.0	-39.6
	**C4-C5 (Å)**	0.43^*b*^	0.25
**25I**	**Δ*θ* (°)**	+36.1^*c*^	-7.6
	**ncBE (kcal mol** ^**-1**^ **)**	-23.6	-35.3

^*a*^ not determined.

^*b*^ in the case of phenylethanol we previously found C4-C4 distances of 0.3 and 0.23 Å for the para- and meta-hydroxylation, respectively.

^*c*^ in the case of phenylethanol we previously found Δ*θ* values of +41° and +22° for the para- and meta-hydroxylation, respectively.

Since we have previously demonstrated that mutations F176A and E103G, removing the hindrance at the “para site”, allow the accommodation of large substituents in para, we have modeled the complexes between the double mutant and the arenium intermediates 24I and 25I. Data in Table 1 and the models in S6 Fig show that the two mutations selectively improve the geometrical parameters calculated for the intermediate 25I. The two mutations also increase the non-covalent binding energy of 25I indicating that this intermediate not only could dock with a better geometry but could bind more tightly to the active site.

**Fig 4:**
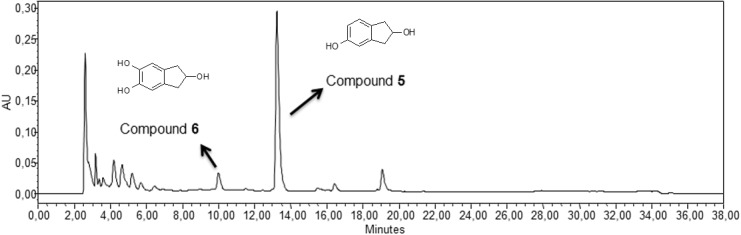
E103G/F176A ToMO-catalyzed biotransformation of 2-indanol. HPLC chromatogram at λ = 280 nm of an aliquot of the exhausted medium deriving from the 16 hrs, E103G/F176A ToMO-catalyzed biotransformation of 2-indanol.

According to the analysis described above, the ToMO variant E103G/F176A was used for the bioconversion of 2-indanol under the same experimental conditions used for the recombinant ToMO *wt* system (Materials and Methods). The HPLC chromatogram in Fig 4 shows in this case the presence of two peaks of products eluting respectively at 13 min (compound **5**, λ_max_ = 280.3 nm) and 10 min (compound **6**, λ_max_ = 288 nm). It is worth noting that the presence of compound **6** was evident only after at least 16h of incubation (Panel C in S2 Fig). Due to a partial loss of viability of *E*.*coli* cells strain JM109 after this time, neither the formation rate nor the yield of this compound were calculated. Rate of formation of compound **5** was of 5.77 (±1.35) μM min^-1^; the yield after 2h of incubation was of 28.9(±0.4)%. By NMR and mass spectrometry analysis (Materials and Methods, and S3 and S4 Figs) compound **5** was identified as 2,5-dihydroxyindan (**DHI**) in agreement with the docking analysis. Moreover, compound **6** was identified as 2,5,6-trihydroxyindan (**THI**, Fig 4 and S4 Fig), thus indicating that in the ToMO variant E103G/F176A the active site is wide enough not only to accommodate 2-indanol in a catalytically productive orientation, but also to permit a second hydroxylation at the position adjacent to the first hydroxyl group, as usually observed when ToMO *wt* acts on its natural substrates toluene and *o*-xylene which are respectively converted to 3-/4-methylcatechol and 4,5-dimethylcatechol [27].

### *In vitro* antioxidant potential of the hydroxylated derivatives of 2-phenoxyethanol, phthalan and 2-indanol

The capacity of the isolated hydroxylated compounds to scavenge the stable radical 2,2-diphenyl-1-picrylhydrazyl (DPPH) was determined spectrophotometrically by measuring the loss of absorbance of DPPH at λ = 515 nm (see Materials and Methods for details). 0.1 mM DPPH and different concentrations of each hydroxylated compound were added, in a final volume of 1mL of methanol, in the range 1–200 μM, and the loss of absorbance at 515 nm (from now on indicated as ABS_515_) was monitored for 30 min. At this time, the percentage of DPPH effectively reduced (%DPPH_red_) was calculated evaluating the initial DPPH concentration at t = 0, indicated as [DPPH]_t = 0_, and the final DPPH concentration at t = 30, referred to as [DPPH]_t = 30_, and using the following formula:

$$\%{\text{DPPH}}_{\text{red}}=[1\text{-}([\text{DPPH}]{}_{\text{t}=30}/[\text{DPPH}]{}_{\text{t}=0})]\;\text{x}\;100$$

The concentration of hydroxylated compound used in each experiment was plotted against %DPPH_red_ (S7 Fig). The concentration that caused a decrease in the initial DPPH concentration of 50% was defined as EC50 and was obtained by a non-linear regression of the experimental curves using the Graphpad Prism software (http://www.graphpad.com/). Each EC50 is the result of at least three independent experiments.

Once obtained the EC50 value for a specific compound we estimated also-at that specific concentration- the time (expressed in minutes) needed to reach a stable value of the ABS_515_, a parameter indicated in literature as TEC50 [37,46]. At this point we calculated what has been defined [46] as the “antiradical efficiency” (AE) a parameter that is expressed by the following formula: AE = 1/(EC50 x TEC50). The AE value, taking into account not only the EC50 but also the reaction time, is more informative than the EC50 value alone on the antioxidant behavior of a compound tested with this specific assay [46].

In all the experiments, described in this and in the following paragraphs, natural aromatic antioxidants such as tyrosol and hydroxytyrosol have also been tested [32–36] as reference molecules, in parallel with the hydroxylated compounds obtained by ToMO-catalyzed biotransformation of 2-phenoxyethanol, phthalan and 2-indanol.

Results are shown in Table 2, where it is evident that some among the phenols and catechols produced by the ToMO-catalyzed reaction on non-natural aromatic substrates show an AE value comparable to the strong natural phenolic antioxidant hydroxytyrosol. Interestingly, compounds such as tyrosol, which has been reported to have antioxidant activity *in vivo* [47], **3HEP, DHiBF,** and **DHI** (S4 Fig) were almost non responsive to the DPPH assay causing negligible losses of ABS_515_ even when used at concentrations up to 2mM (data not shown).

**Table 2:** Evaluation of the antioxidant activity using the DPPH assay.

Compound	EC50 (μM Antioxidant/μM DPPH)	TEC50 (min)	AE
Hydroxytyrosol	0.21 (±0.01)	18.0 (±2.0)	**0.27** (±0.01)
4HEP	0.40 (±0.04)	19.0 (±1.0)	**0.140** (±0.006)
2HEP	1.00 (±0.02)	25.3 (±2.1)	**0.040** (±0.002)
THI	0.40 (±0.01)	15.3 (±2.5)	**0.15** (±0.03)

EC50, TEC50 and antioxidant efficiencies (AE) values of hydroxytyrosol, 4 HEP, 2HEP and THI. Standard deviations (SD) are reported in parentheses.

DPPH reduction curves obtained for each compound tested (S7 Fig) and the AE values calculated (Table 2) clearly demonstrate the relevant reducing potential of **2HEP**, **4HEP** and of **THI** compared to hydroxytyrosol activity. Although this could be expected in the case of **THI**, as it shares a catechol moiety with the hydroxytyrosol structure, results obtained from **2HEP** and **4HEP** (phenols like tyrosol, **DHiBF,** and **DHI)** deserve some comments. In both structures the aromatic ring hosts two oxygens either in ortho (**2HEP**) or para (**4HEP**); thus these two molecules share an important structural similarity with catechol and hydroquinone, respectively, two known antioxidants [48]. **3HEP**, instead, shows the additional atom oxygen in a meta position respect to the-OH already present on the aromatic ring, thus resembling resorcinol. Compared to catechol and hydroquinone, where the-OH group in either ortho or para position has an electron donating effect, the reaction of resorcinol with a radical such as DPPH would theoretically be reduced by the electron-withdrawing effect of the-OH group in the meta position [9]. The same rational seems to apply to the three hydroxylated derivatives we obtained from the biotransformation of 2-phenoxyethanol.

Interestingly, the **THI** plot shows a sigmoidal profile that was not observed for the other compounds tested. Although a more detailed investigation would be necessary to account for such a behaviour, it may be argued that a two-step oxidation path is available for **THI** as a consequence of the-OH substituted five member ring condensed with the catechol system (S8 Fig and explicative notes).

### Antioxidant potential of 4HEP, 3HEP, 2HEP, DHiBF, DHI, THI in cultured H9c2 cells

The potential antioxidant activity of the hydroxylated compounds described in this study was also evaluated testing their effect in a eukaryotic cell system such as the rat cardiomyoblast cell line H9c2. These latter are non-malignant cardiac-like cells, commonly used to study the molecular response to oxidative damage [39–40]. We first examined the effect of the hydroxylated compounds on cell viability, by using the MTT assay (Panel A in S9 Fig), to verify that a cytotoxic effect did not occur. To this purpose, cells were cultured for 72 hours under normal growth conditions in the presence of each purified hydroxylated compound, previously diluted in the growth medium at optimized concentrations (Materials and Methods). The percentage of viable cells, compared to the control, never significantly decreased as shown in Panel A in S9 Fig, thus showing that the compounds tested are not cytotoxic on this specific cell line. Indeed, in the presence of the non-hydroxylated precursors (Panel A in S9 Fig, dark white bars) used as substrates in the ToMO-catalyzed biotransformations, the percentage of viable cells was almost close to the 100%. It is worth to note that hydroxytyrosol and tyrosol induced a significant increase of cell survival (Panel A in S9 Fig, black bars) (*p* value < 0.01). A slight positive effect on cell viability was also observed when using the hydroxylated products obtained from 2-phenoxyethanol, with a significant increase of the viable cells number (*p* value < 0.01 for **4HEP**, *p* value < 0.05 for **3HEP** and **2HEP**). Conversely, **DHiBF**, **DHI** and **THI** did not produce any apparent change in cell viability.

Optimized concentrations obtained in this assay were used in all subsequent experiments (Materials and Methods). In order to validate the data obtained on cell viability, we investigated whether the tested compounds had any effect on cell apoptosis. Apoptotic cells were identified by acridine orange (AO) and ethidium bromide (EB) staining of nuclei [45], as described in Materials and Methods; results are presented in Panel B and C in S9 Fig When cells were cultured under normal growth conditions, the hydroxylated compounds did not induce an increase in cell apoptosis (Panel C in S9 Fig, white bars). This is consistent with the results obtained from the MTT assay. Moreover, tyrosol, **3HEP**, **DHI** and **THI** induced a significant decrease of the number of apoptotic cells (*p* value < 0.05 for **DHI**, *p* value ≤ 0.01 for the other compounds; Panel C in S9 Fig). Exposure of H9c2 to 0.25 mM of SA for 90 min induced, *per se*, a 37% increase in the percentage of apoptotic nuclei (Panel C in S9 Fig, first black bar). Since this stress condition was sufficient to elicit a detectable but still sub-lethal cell stress response, it was considered suitable for further experiments aimed at investigating the potential protective effect of our compounds towards the SA-induced effect. Pretreatment for 3h with **4HEP**, **2HEP**, **DHI** and **THI** resulted in a significant reduced percentage of apoptotic nuclei, which decreased from 37% of the control to around 20% (Panel C in S9 Fig, black bars) (*p* value < 0.05 for **2HEP** and **DHI**, ≤ 0.01 for **4HEP** and **THI**). The other hydroxylated compounds did not significantly alter the sodium arsenite (SA)-induced apoptotic effect.

**Fig 5:**
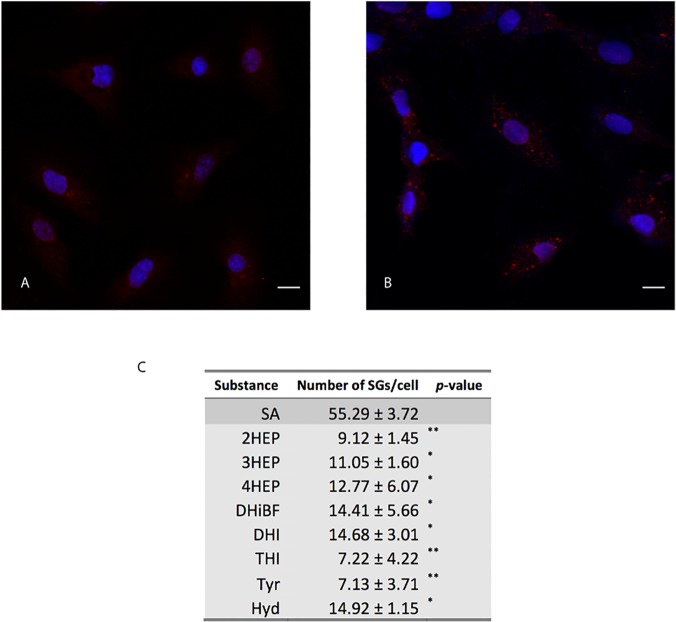
Immunofluorescence assay. Effect of sodium arsenite on the formation of stress granules: immunofluorescence and statistical analysis. PABP mAb and Cy3-conjugated goat anti-mouse F(ab’)_2_ were used to detect SGs in H9c2 cells cultured under normal growth **(A)** and SA-stress conditions **(B)**. Nuclei are stained with DAPI. Scale bars: 10μm. **(C)** Effect of pretreatment with hydroxylated compounds in SA-treated H92c cardiomyoblasts. The table reports SGs number for cell (Mean ± SEM) that was obtained by using “Analyze Particles” plugin of the image software ImageJ (pixel unit: 1,5–2,0; Circularity: 0,99–1,00). Statistical analysis (unpaired t test, two tailed; (*, p ≤ 0.05; **, p ≤ 0.01). Tyr: tyrosol; Hyd: hydroxytyrosol.

To better assess a potential antioxidant activity of our hydroxylated compounds, a cell stress response was analyzed, which is activated under mild stress conditions when a transient translational arrest occurs. As underlined in the Introduction section, exposure to SA inhibits protein synthesis and activates multiple stress signaling pathways, including the formation of stress granules (SGs) [4,41–43]. SGs are cytoplasmic foci composed of 40S ribosomal subunits, initiation translation factors and their associated mRNA transcripts, which aggregate when translation initiation is stalled. The SGs formation is reversible and the protein synthesis can be restored, if the stress condition is recovered. To investigate the potential antioxidant effect of our hydroxylated compounds, we examined the occurrence and the number of stress granules (SGs) in H9c2 cells both under normal growth and SA-induced stress conditions. SGs were detected by immunofluorescence and confocal microscopy analysis using the marker PABP (poly-A binding protein) (Materials and Methods and Fig 5). To estimate the antioxidant activity, SGs number per cell was calculated by using a statistical analysis, applied to at least three images acquired analyzing a minimum of 350–400 cells. When H9c2 cells were cultured under normal growth conditions, in the presence of the hydroxylated compounds, no significant change in SGs formation was observed (11.7 ± 4.4) when compared to the control, confirming that the compounds tested do not alter the cellular status (data not shown). In SA-treated cells, SGs were instead easily identified and the statistical analysis revealed the presence of 55.3 ± 3.7 SGs per cell (Fig 5). When pre-treatment with the hydroxylated compounds was performed before the SA-induced oxidative stress, a strong and statistically significant decrease in SGs number was observed (Fig 5). **2HEP** and **THI**, again, showed a higher antioxidant activity compared to the other hydroxylated compounds. A putative antioxidant activity of the aromatic non-hydroxylated substrates used was also evaluated. Their effect on SGs in stressed H9c2 cells was analyzed (data not shown), and no significant decrease in SGs number was observed, when compared to the control.

## Conclusions

Aromatic nuclei are, for their chemical versatility, ideal candidates to create molecules with multiple pharmacological effects. Phenols and catechols, in particular, are promising starting points to merge with other pharmacophores thus obtaining multipotent agents with antioxidant effects and beyond. The different studies presented on the ToMO multicomponent system in more than a decade [18,20–21,23–31] described the unique versatility of the catalytic activity of this enzyme, which allows to obtain hydroxylated derivatives from a wide array of aromatic substrates, highlighting its role as a potential resource for the biosynthesis of phenolic and catecholic compounds.

We have previously shown that a fine-tuning of the regioselectivity and of the catalytic efficiency of ToMO on 2-phenylethanol, a non-natural substrate, to produce known antioxidants such as tyrosol and hydroxytyrosol could be achieved by using an optimized computational model that allows a careful alteration of the shape of the active site [26,18].

In this work we investigated the use of ToMO for the biosynthesis of six aromatic antioxidants, starting from three commercially available aromatic compounds that are non-natural substrates of the enzyme. By using either the *wt* enzyme or ToMO mutant E103G/F176A-catalyzed biotransformations, six hydroxylated compounds were obtained. Although not all of them were able to reduce the DPPH stable radical, a common test system for determining the scavenging properties of putative antioxidants *in vitro*, all hydroxylated compounds shared a common, although differential, antioxidant effect on eukaryotic cells of the H9c2 cell line subjected to a mild oxidative stress induced by sodium arsenite. The discrepancy between the two assays showed from one side a possible limitation in the use of the DPPH assay as the unique methodology to assess the *in vitro* antioxidant potential of hydroxylated aromatic compounds, and on the other hand confirms that the antioxidant effect of phenols and catechols frequently observed in cells might not be due to a simple scavenge of free radicals, but might be dependent on other molecular events that have not been completely elucidated yet.

The availability of the molecules produced under the present study undoubtedly opens new possibilities for obtaining a whole new set of antioxidant molecules by using the ToMO multicomponent system as biocatalyst using a wide array of commercially available monocyclic aromatic compounds as starting substrates.

## Supporting Information

S1 Fig Bioconversion of 2-phenylethanol. Natural antioxidants tyrosol and hydroxytyrosol produced from the hydroxylation of 2-phenylethanol catalyzed by ToMO mutants E103G/F176T and E103G/F176I (Notomista E., Scognamiglio R., Troncone L., Donadio G., Pezzella A., Di Donato A., and Izzo V. Tuning the specificity of the recombinant multicomponent toluene o-xylene monooxygenase from Pseudomonas sp. strain OX1 for the biosynthesis of tyrosol from 2-phenylethanol. Appl. Environ. Microbiol. 2011.77(15): 5428–37. http://aem.asm.org/content/77/15/5428.full).
(TIF)

S2 Fig Time course chromatograms. Time course formation of the hydroxylated products deriving from the ToMO-catalyzed bioconversion of: **(Panel A)** 2-phenoxyethanol, **(Panel B)** phthalan and **(Panel C)** 2-indanol. Chromatograms were extracted at λ = 280 nm. The 2-indanol bioconversion was performed by using ToMO mutant E103G/F176A.
(TIF)

S3 Fig NMR analysis. NMR analysis of the isolated products deriving from the ToMO-catalyzed bioconversion of: 2-phenoxyethanol (Compounds **1**, **2** and **3**), phthalan (Compound **4**) and 2-indanol (Compounds **5** and **6**). The structures deduced for each new compound obtained are also presented.
(TIF)

S4 Fig Mass spectrometry analysis. Mass spectrometry analysis and molecular weights obtained for the isolated products deriving from the ToMO-catalyzed bioconversion of: 2-phenoxyethanol (Compounds **1**, **2** and **3**), phthalan (Compound **4**) and 2-indanol (Compounds **5** and **6**).
(TIF)

S5 Fig Estimation of extinction coefficients. For each hydroxylated compound isolated, the εat λ_max_ of a similar model compound is reported which was used for the determination of the concentration throughout this study.
(TIF)

S6 Fig Modelling. Monte Carlo minimized models of the complexes between ToMO or (E103G,F176A)-ToMO and the arenium intermediates for the toluene/p-cresol (S4M) and the 2-indanol/2,5-dihydroxyindan (25I) reactions. Carbon atoms are shown in green (ToMO/S4M), magenta (ToMO/25I) and yellow [(E103G,F176A)-ToMO/25I]. Oxygen atoms are shown in red, nitrogen atoms in blue, sulphur atoms in dark yellow. Hydrogen atoms are not shown. Iron ions are shown as orange spheres.
(TIF)

S7 Fig DPPH assay. DPPH reduction (expressed as the percentage of the DPPH effectively reduced, Y axis) as a function of the μM concentration of the compound tested (X axis). 4-(2-Hydroxyethoxy)phenol **(4HEP)**; 2-(2-Hydroxyethoxy)phenol **(2HEP)**; 2,5,6-trihydroxyindan (**THI**); Hydroxytyrosol.
(TIF)

S8 Fig Oxidation pathway. Two-step oxidation pathway proposed for THI as a consequence of its peculiar structure showing an-OH substituted five member ring condensed with the catechol moiety. First oxidation step results in the formation of expected orthoquinone moiety which can itself undergoes tautomerization affording-via transient quinone methides- a cyclopentanone condensed catechol, again susceptible to act as reductant (Pezzella A., Lista L., Napolitano A., and d’Ischia M. Tyrosinase-catalyzed oxidation of 17beta-estradiol: structure elucidation of the products formed beyond catechol estrogen quinones. Chem.Res. Toxicol. 2005.18(9): 1413–9. http://pubs.acs.org/doi/abs/10.1021/tx050060o).
(TIF)

S9 Fig MTT viability assay and apoptosis assay. **(Panel A)** H9c2 cells were cultured under normal growth conditions, incubated in the presence of either the substrates (dark white bars) or the hydroxylated derivatives (black bars) for 72 hours. Cell viability was determined by means of ABS_570_ compared to the control (white bar), that is cells without any treatment. Data shown are the means ± s.d of at least three repeats, of a representative experiment. A statistical analysis by two-tailed Student’s t was performed. **(Panel B)** and **(Panel C)** Acridine orange (AO) and ethidium bromide (EB) staining of nuclei to identify apoptotic nuclei. H9c2 cells were cultured under either normal growth conditions (image on the left, panel B, and white bars in panel C), or SA-induced oxidative stress (image on the right, panel B, and black bars in panel C).
(TIF)
