# Supplementary figures and images for: The Toluene o-Xylene Monooxygenase Enzymatic Activity for the Biosynthesis of Aromatic Antioxidants
Source: PLoS One. 2015 Apr 27;10(4):e0124427. doi: 10.1371/journal.pone.0124427 (PMC4411060; doi:10.1371/journal.pone.0124427)

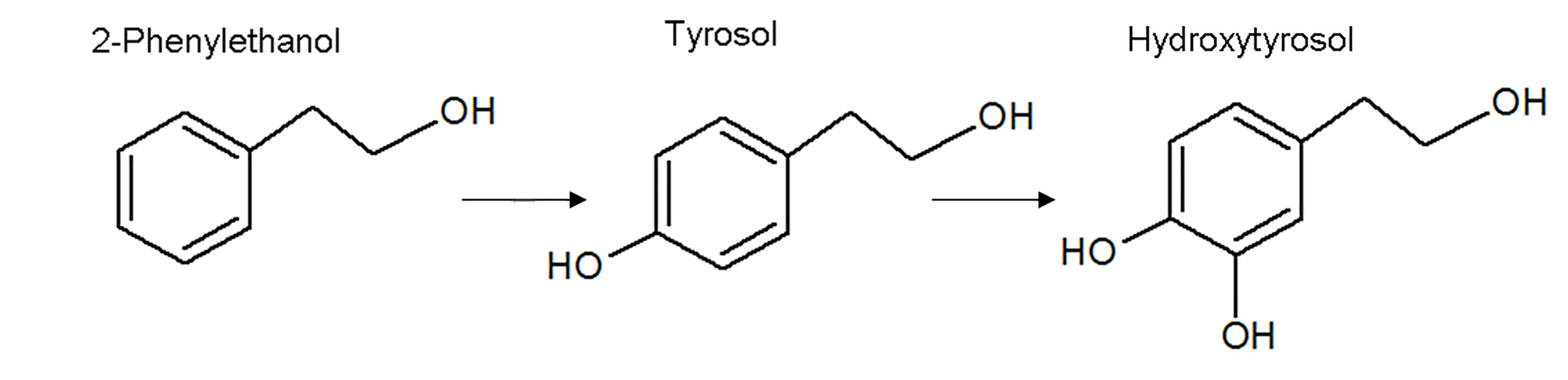

Supplement: S1 Fig — Natural antioxidants tyrosol and hydroxytyrosol produced from the hydroxylation of 2-phenylethanol catalyzed by ToMO mutants E103G/F176T and E103G/F176I (Notomista E., Scognamiglio R., Troncone L., Donadio G., Pezzella A., Di Donato A., and Izzo V. Tuning the specificity of the recombinant multicomponent toluene o-xylene monooxygenase from Pseudomonas sp. strain OX1 for the biosynthesis of tyrosol from 2-phenylethanol. Appl. Environ. Microbiol. 2011.77(15): 5428–37. http://aem.asm.org/content/77/15/5428.full). (TIF) [file pone.0124427.s001.tif]

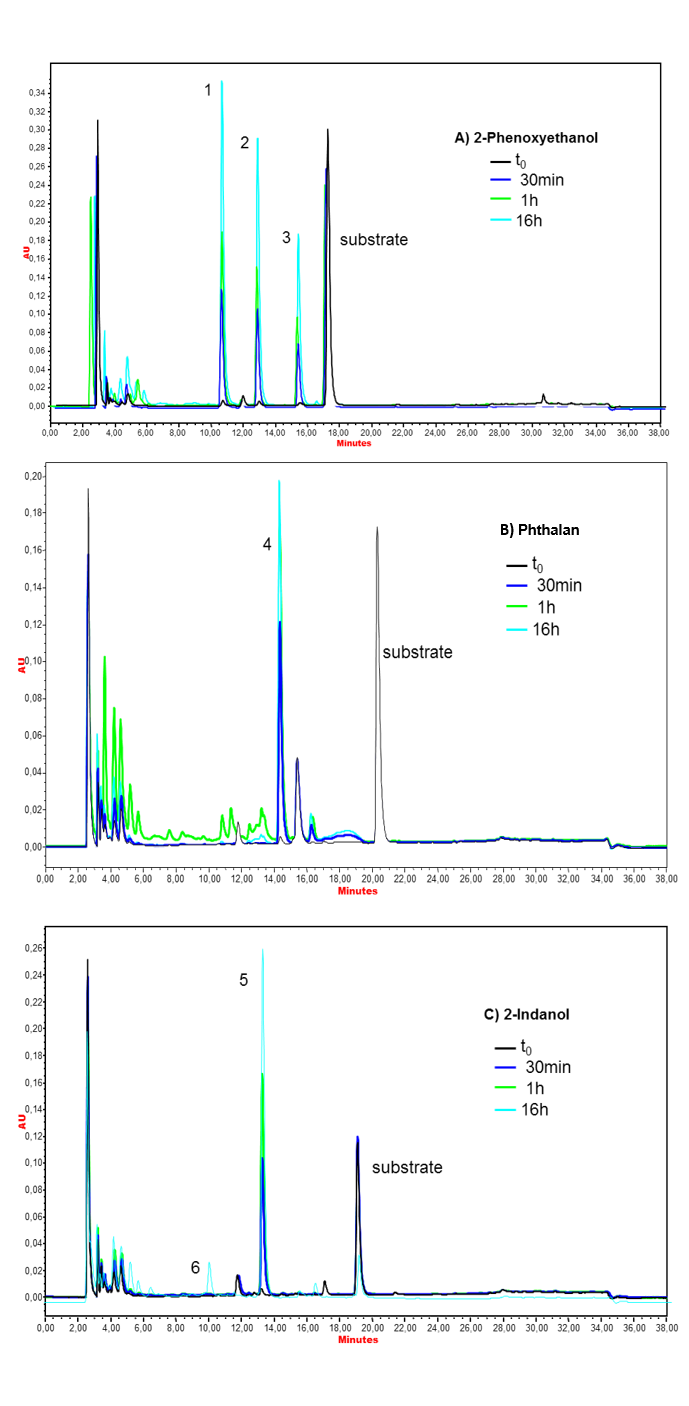

Supplement: S2 Fig — Time course formation of the hydroxylated products deriving from the ToMO-catalyzed bioconversion of: (Panel A) 2-phenoxyethanol, (Panel B) phthalan and (Panel C) 2-indanol. Chromatograms were extracted at λ = 280 nm. The 2-indanol bioconversion was performed by using ToMO mutant E103G/F176A. (TIF) [file pone.0124427.s002.tif]

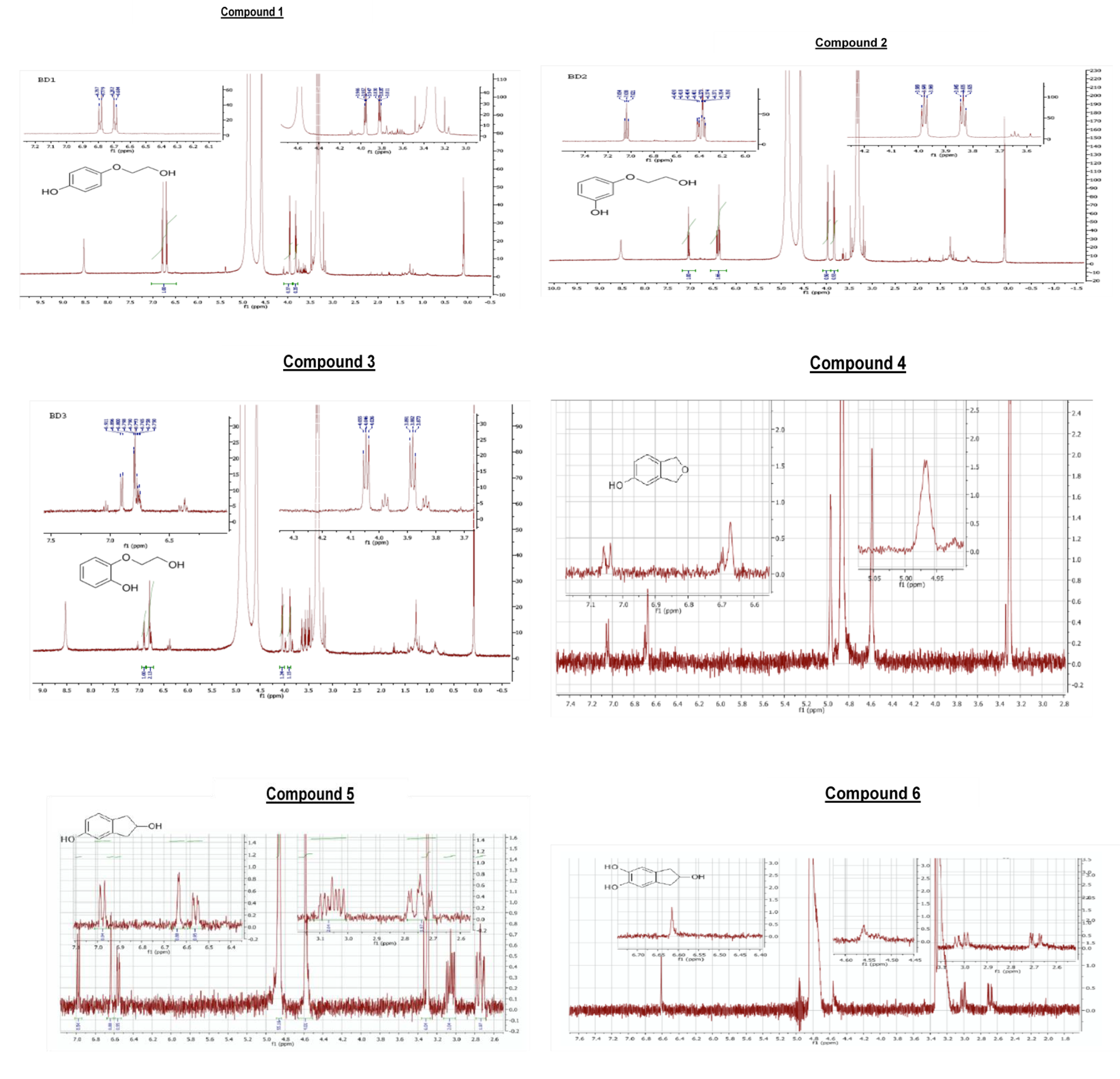

Supplement: S3 Fig — NMR analysis of the isolated products deriving from the ToMO-catalyzed bioconversion of: 2-phenoxyethanol (Compounds 1, 2 and 3), phthalan (Compound 4) and 2-indanol (Compounds 5 and 6). The structures deduced for each new compound obtained are also presented. (TIF) [file pone.0124427.s003.tif]

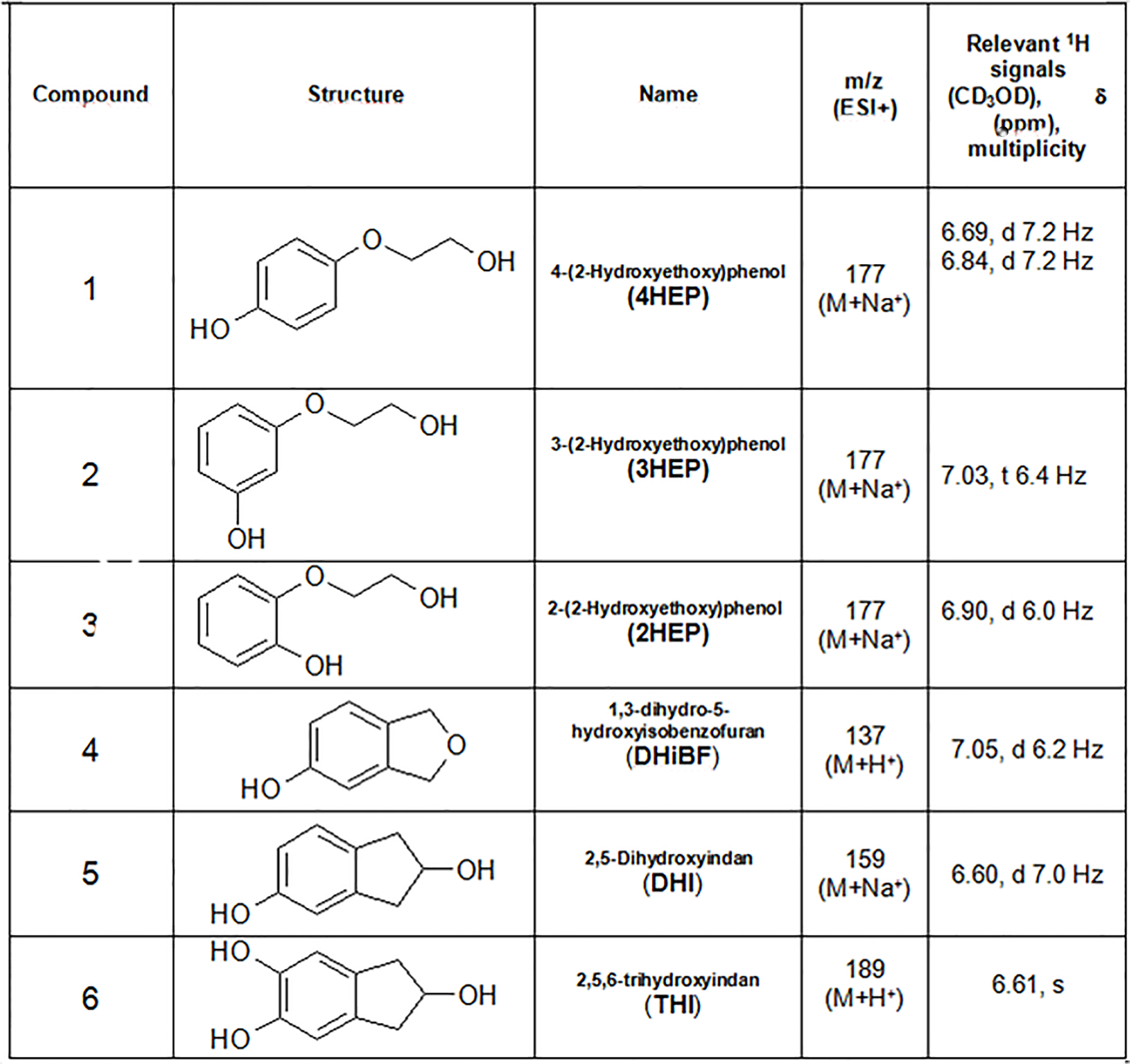

Supplement: S4 Fig — Mass spectrometry analysis and molecular weights obtained for the isolated products deriving from the ToMO-catalyzed bioconversion of: 2-phenoxyethanol (Compounds 1, 2 and 3), phthalan (Compound 4) and 2-indanol (Compounds 5 and 6). (TIF) [file pone.0124427.s004.tif]

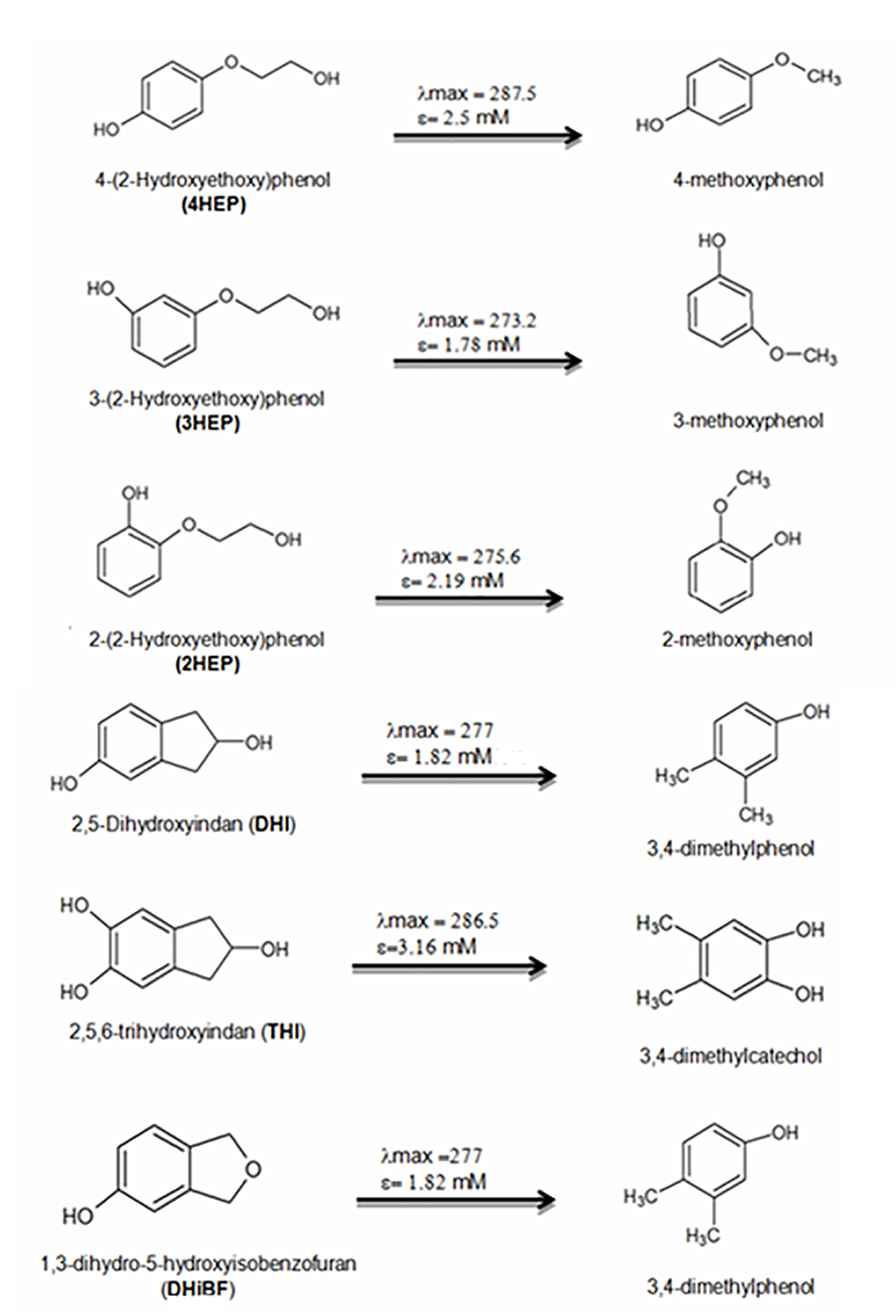

Supplement: S5 Fig — For each hydroxylated compound isolated, the εat λmax of a similar model compound is reported which was used for the determination of the concentration throughout this study. (TIF) [file pone.0124427.s005.tif]

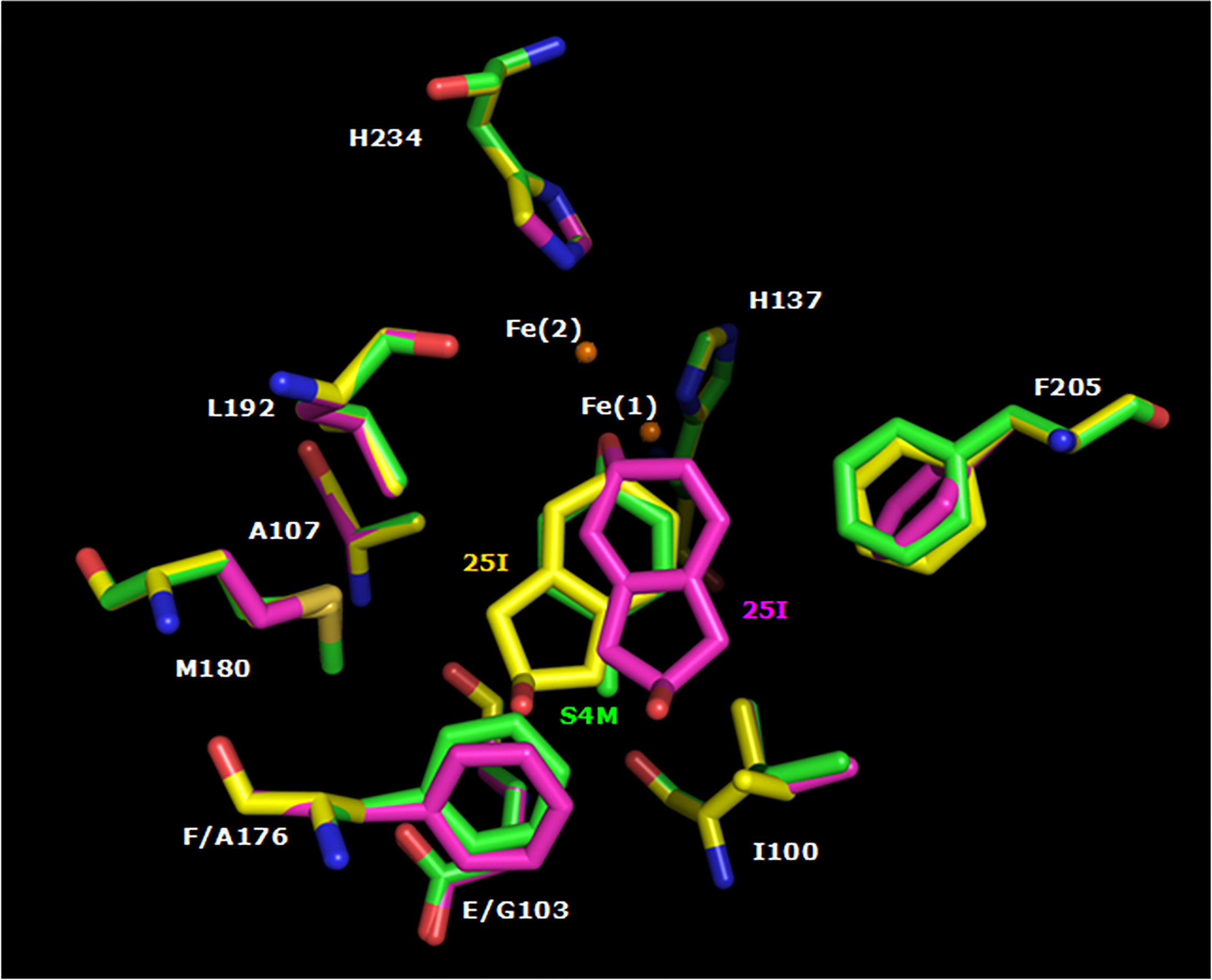

Supplement: S6 Fig — Monte Carlo minimized models of the complexes between ToMO or (E103G,F176A)-ToMO and the arenium intermediates for the toluene/p-cresol (S4M) and the 2-indanol/2,5-dihydroxyindan (25I) reactions. Carbon atoms are shown in green (ToMO/S4M), magenta (ToMO/25I) and yellow [(E103G,F176A)-ToMO/25I]. Oxygen atoms are shown in red, nitrogen atoms in blue, sulphur atoms in dark yellow. Hydrogen atoms are not shown. Iron ions are shown as orange spheres. (TIF) [file pone.0124427.s006.tif]

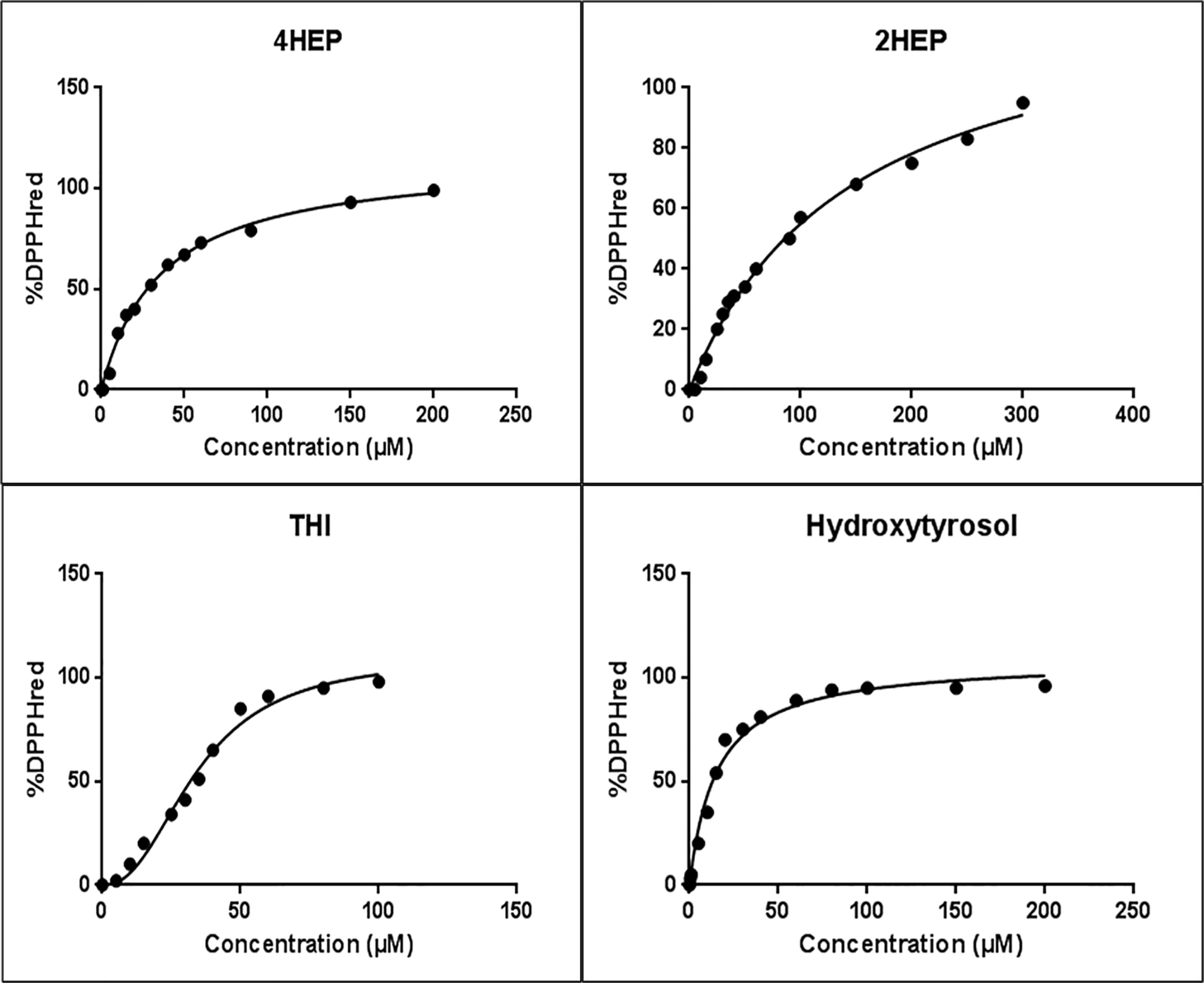

Supplement: S7 Fig — DPPH reduction (expressed as the percentage of the DPPH effectively reduced, Y axis) as a function of the μM concentration of the compound tested (X axis). 4-(2-Hydroxyethoxy)phenol (4HEP); 2-(2-Hydroxyethoxy)phenol (2HEP); 2,5,6-trihydroxyindan (THI); Hydroxytyrosol. (TIF) [file pone.0124427.s007.tif]

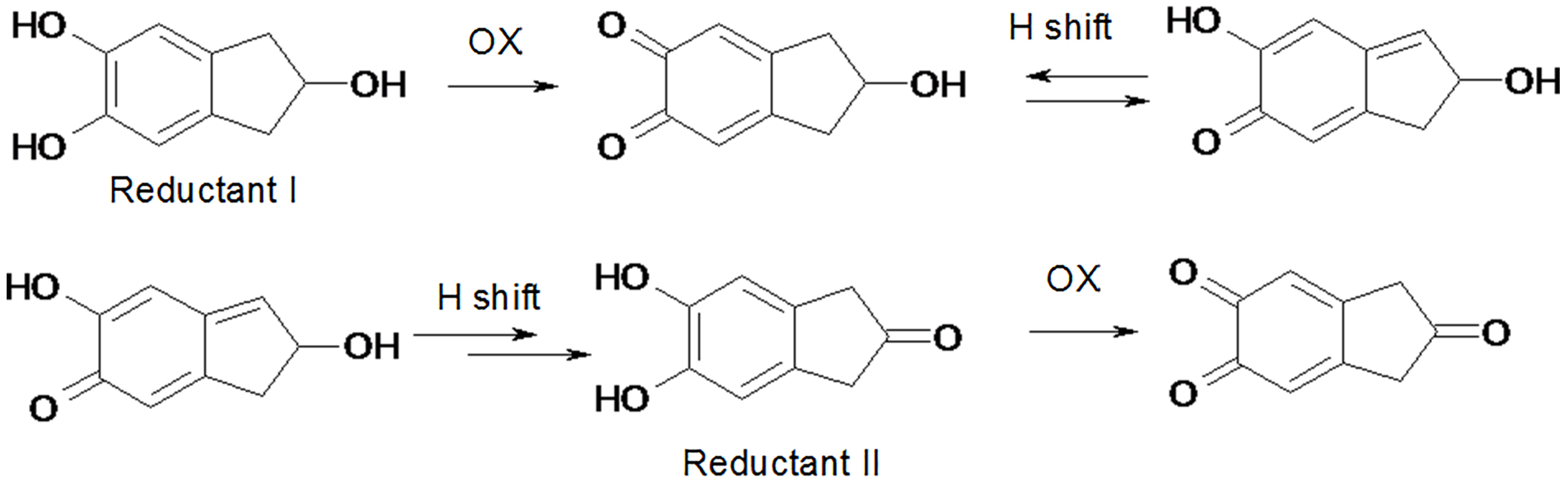

Supplement: S8 Fig — Two-step oxidation pathway proposed for THI as a consequence of its peculiar structure showing an-OH substituted five member ring condensed with the catechol moiety. First oxidation step results in the formation of expected orthoquinone moiety which can itself undergoes tautomerization affording-via transient quinone methides- a cyclopentanone condensed catechol, again susceptible to act as reductant (Pezzella A., Lista L., Napolitano A., and d’Ischia M. Tyrosinase-catalyzed oxidation of 17beta-estradiol: structure elucidation of the products formed beyond catechol estrogen quinones. Chem.Res. Toxicol. 2005.18(9): 1413–9. http://pubs.acs.org/doi/abs/10.1021/tx050060o). (TIF) [file pone.0124427.s008.tif]

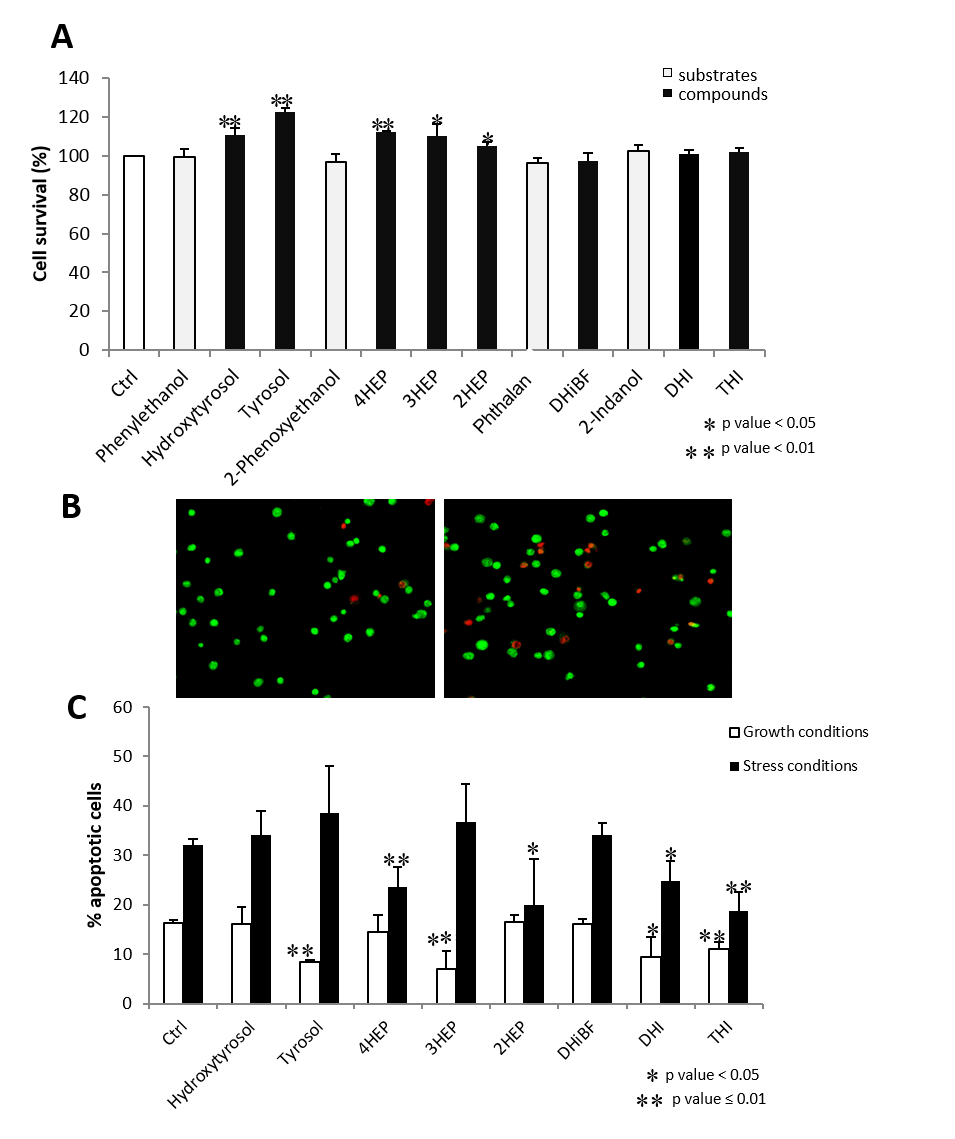

Supplement: S9 Fig — (Panel A) H9c2 cells were cultured under normal growth conditions, incubated in the presence of either the substrates (dark white bars) or the hydroxylated derivatives (black bars) for 72 hours. Cell viability was determined by means of ABS570 compared to the control (white bar), that is cells without any treatment. Data shown are the means ± s.d of at least three repeats, of a representative experiment. A statistical analysis by two-tailed Student’s t was performed. (Panel B) and (Panel C) Acridine orange (AO) and ethidium bromide (EB) staining of nuclei to identify apoptotic nuclei. H9c2 cells were cultured under either normal growth conditions (image on the left, panel B, and white bars in panel C), or SA-induced oxidative stress (image on the right, panel B, and black bars in panel C). (TIF) [file pone.0124427.s009.tif]
